# Paeoniflorin, a Natural Product With Multiple Targets in Liver Diseases—A Mini Review

**DOI:** 10.3389/fphar.2020.00531

**Published:** 2020-04-28

**Authors:** Xiao Ma, Wenwen Zhang, Yinxiao Jiang, Jianxia Wen, Shizhang Wei, Yanling Zhao

**Affiliations:** ^1^School of Pharmacy, Chengdu University of Traditional Chinese Medicine, Chengdu, China; ^2^Department of Pharmacy, Fifth Medical Center of PLA General Hospital, Beijing, China

**Keywords:** paeoniflorin, hepatic protection, cholestasis, liver fibrosis, nonalcoholic fatty liver disease, hepatocellular carcinoma, mini-review

## Abstract

Paeoniflorin is derived from *Paeonia suffruticosa* Andr., *Paeonia lactiflora* Pall., or *Paeonia veitchii* Lynch and has been used in traditional medical applications for more than 2,000 years. Paeoniflorin is a monoterpenoid glycoside with various effects on liver diseases. Recent studies have revealed that paeoniflorin demonstrates a wide range of activities, including hepatic protection, cholestasis alleviation, liver fibrosis attenuation, nonalcoholic fatty liver disease prevention, and hepatocellular carcinoma inhibition involved in multiple pathways. Moreover, anti-inflammation, antioxidation, and immune regulation with the regulation of TLR4-NF-κB, ROCK/NF-κB, HO-1, mitochondria-dependent as well as HMGB1‐TLR4 signaling pathways are correlated with hepatic protection in liver injury and nonalcoholic fatty liver disease. Antioxidative mechanisms, anti-inflammation, and hepatic transporter regulation involved in NOX4, PI3K/Akt/Nrf2, NF‐κB, NTCP, BSEP, as well as MRP2 signals are mainly relevant to the anticholestatic effect of paeoniflorin. The inhibition of hepatic stellate cell activation and alleviation of extracellular matrix deposition *via* vast signals such as mTOR/HIF-1α, TGF-β1/Smads, and JAK2/STAT6 are primarily involved in the antifibrotic effect of paeoniflorin. The regulation of macrophages also contributes to the alleviation effect on liver fibrosis. In addition, the reduction of invasion, metastasis, and adhesion and the induction of apoptosis-related targets, including Bax, Bcl-2, and caspase-3, are related to its effect on hepatocellular carcinoma. The literature indicates that paeoniflorin might have potent efficacy in complex liver diseases and demonstrates the profound medicinal value of paeoniflorin.

## Introduction

Since the “one gene, one drug, one disease” concept was challenged, more and more agents have been confirmed to be multiple targets and signals rather than a single approach (Hasin et al., 2017). Several natural products applied for thousands of years in traditional medicine demonstrate a wide range of pharmacological activities *via* multiple pathways (Park and Pezzuto, 2017; Kunnumakkara et al., 2017; Ren et al., 2019). These natural products might have potent efficacy in complex human diseases and display profound medicinal value. Among these natural compounds, paeoniflorin has gained attention as a promising compound for drug development.

Paeoniflorin is the major bioactive ingredient derived from *Paeonia suffruticosa* Andr., *Paeonia lactiflora* Pall., or *Paeonia veitchii* Lynch, which have been used for cerebrovascular disease, cardiovascular disease, nervous system disease, and liver disease in traditional Chinese medicine for more than 2,000 years (Zhao et al., 2016). Paeoniflorin was first isolated from *Paeonia lactiflora* Pall. as a monoterpenoid glycoside in 1963 (Zhang J. et al., 2018). Since then, an increasing number of studies have reported the numerous pharmacologic effects of paeoniflorin, such as cerebrovascular protection, cardiovascular protection, neuroprotection, antihyperglycemia, tumor inhibition, immunoregulation, abirritation, and hepatoprotection (Zhang et al., 2017; Chen et al., 2018; Zhai et al., 2018; Tu et al., 2019). Paeoniflorin has gained a large amount of attention for its effect on liver diseases as the growth rate of liver diseases has increased in recent years (Ma et al., 2020). Hence, this mini-review provides a comprehensive summary of the pharmacologic activities of paeoniflorin in liver diseases.

## Hepatic Protection

The liver is a vital organ for metabolic functions and for the purification of toxic chemicals. However, the liver can be overloaded (Singh et al., 2016). Once the function of the liver is dysregulated, liver damage will occur. Under certain circumstances, liver injuries can be induced by various factors, including chemical pollutants, drugs, alcohols, and liver ischemia (Peralta et al., 2013; Wang et al., 2016; Kullak-Ublick et al., 2017). Liver injury is recognized as a highly complex process accompanied by extensive apoptosis in hepatic cells. Oxidative stress and inflammatory reactions are thought to play key roles in this process (Brenner et al., 2013; Li et al., 2015). Moreover, immune reactions resulting from immune cells such as Kupffer cells have also drawn much attention due to the unique characteristics of hepatic sinusoids (Heymann et al., 2015).

### Hepatic Ischemia/Reperfusion Alleviation

Hepatic ischemia/reperfusion (I/R) injury is the major manifestation after liver transplantation or hemorrhagic shock with relatively high morbidity and mortality (Go et al., 2015). Currently, paeoniflorin is considered to be highly effective in hepatic I/R injury treatment. A study from Xie reported that compared with hepatic I/R injury rats, rats pretreated with paeoniflorin (100 mg/kg) showed significantly decreased serum alanine aminotransferase (ALT) and aspartate aminotransferase (AST) activities by 40.3% and 53.8%, respectively. This liver protection effect is strongly relevant to directly alleviating hepatic cell apoptosis and decreasing caspase-3 levels. Furthermore, an inflammatory response was also observed in this study. This study demonstrated that paeoniflorin pretreatment could inhibit CD45+/Ly6G+ neutrophils and the production of proinflammatory cytokines [tumor necrosis factor alpha (TNF-α) and interleukin-1 beta (IL-1β)]. Therefore, a reduction in the innate immune response might contribute to this process. This research finally found that inhibiting the HMGB1‐TLR4 signaling pathway is the crucial mechanism of paeoniflorin in hepatic I/R injury (Xie et al., 2018). Another study also supported this conclusion. Tao's study demonstrated that treatment with paeoniflorin at a dose of 5 to 20 mg/kg could markedly reduce the expression levels of inflammatory mediators, including nuclear factor kappa-B (NF‐κB), TNF-α, IL-1β, and IL-6. At the same time, the apoptosis marker caspase‐3 was decreased after paeoniflorin treatment (Tao et al., 2016). Therefore, antioxidative, anti‐inflammatory, and antiapoptotic activities are clearly involved in the mechanism of paeoniflorin treatment.

### Protection From Toxic Chemical-Induced Liver Injury

There are a variety of toxic chemicals, including carbon tetrachloride (CCl_4_), concanavalin A (Con-A), D-galactosamine (GalN), bacillus Calmette-Guérin (BCG), and lipopolysaccharide (LPS), that can induce liver injury (Zhang H.Y. et al., 2018). Toxic chemical-induced liver injury is mainly characterized by an immune response and inflammation. Moreover, hepatic tissue apoptosis is a key outcome. A large amount of evidence indicates that paeoniflorin has a profound effect on toxic chemical-induced liver injury. Paeoniflorin at a dose of 100 mg/kg was able to decrease liver injury, significantly decrease ALT and AST, and alleviate the histopathological changes induced by CCl_4_. Moreover, the significant pharmacological effect was related to the reduction of HO-1 mRNA expression and proinflammatory cytokine (TNF-α and IL-6) excretion (Guo et al., 2018). Other studies have indicated the protective effect of paeoniflorin on Con A-induced hepatitis with immune regulation. Chen's results suggested that intravenous paeoniflorin pretreatment could attenuate plasma levels of ALT and AST and diminish apoptosis or necrosis of liver tissue. These results demonstrated a protective effect of paeoniflorin against Con A-induced liver injury in mice. The mechanism may at least in part be the suppression of CD4+, CD8+, and NKT cell infiltration in the liver. Moreover, the downregulation of TLR4 expression and the inhibition of NF-κB activation are key signaling pathways in this process (Chen M. et al., 2015). *In vitro* research from primary human hepatic sinusoidal endothelial cells (HHSECs) also confirmed this result. Paeoniflorin at doses from 50 to 800 μM most likely contributed to the alleviation of Con-A-induced inflammation in HHSECs. Preincubation with paeoniflorin caused a concentration-dependent downregulation of IL-8. Furthermore, paeoniflorin was able to inhibit IL-8 release by 52.8% at a dose of 800 μM. The mechanism might be closely related to blocking IL-8 secretion *via* the downregulation of ERK1/2 and Akt phosphorylation (Gong et al., 2015). In addition, GalN/TNF-α-induced apoptosis of human L-02 hepatocytes was decreased by paeoniflorin in a dose-dependent manner. The antiapoptotic effect was further evidenced by the inhibition of caspase-3/9 activities and by the suppression of ER stress activation in L-02 cells. These results revealed that paeoniflorin might target ER stress and calcium, leading to mitochondria-dependent pathway regulation (Jiang et al., 2014). A study of immunological liver injury based on BCG combined with LPS was also performed in 2006. Paeoniflorin administration was able to protect against immunological liver injury by ameliorating TNF-α and IL-6 secretion and downregulating LPS receptor expression (Liu et al., 2006).

## Cholestasis Alleviation

Cholestasis is characterized by decreased bile flow and bile acid accumulation. It is one of the most common but devastating liver diseases. Hepatocyte injury and cholangitis will ultimately occur with cholestasis progression. Furthermore, portal myofibroblast and hepatic stellate cell activation rapidly result in biliary fibrosis or even cirrhosis without prompt treatment (Ghonem et al., 2015). It is currently believed that the pathogenesis of cholestasis involves multiple signaling pathways with the simultaneous activation of inflammation, dysregulation of hepatocyte transporters, and oxidative stress injury in liver tissue (Copple et al., 2010; Allen et al., 2011; Trauner et al., 2017).

A series of studies from Zhao's group indicated that paeoniflorin exerts a dose-dependent (50–200 mg/kg) protective effect on alpha-naphthylisothiocyanate (ANIT)-induced cholestasis in rats by decreasing serum ALT, AST, TBIL, DBIL, total bile acid (TBA), γ-glutamyltranspeptidase (γ-GT), and alkaline phosphatase (ALP). Moreover, the extremely suppressed bile flow induced by ANIT was also increased by paeoniflorin treatment. The mechanism of this activity is partially related to attenuating oxidative stress with reactive oxygen species (ROS) inhibition by suppressing nicotinamide adenine dinucleotide phosphate (NADPH) oxidase 4 expression and the mitochondria-dependent pathway (Zhao et al., 2013; Zhou et al., 2017). In addition, an alternative antioxidative mechanism was also investigated. The results indicated that paeoniflorin could regulate glutathione (GSH) and its related synthase glutamate-cysteine ligase catalytic subunit (GCLc) and glutamate-cysteine ligase modifier subunit (GCLm). The enhancement of GSH synthesis was further proven to increase Nrf2 through the PI3K/Akt-dependent pathway (Chen Z. et al., 2015). Regarding inflammation, histological examination revealed that paeoniflorin-treated rats demonstrated less neutrophil infiltration. The research suggested that paeoniflorin could remarkably reduce the overexpression of NF-κB and IL-1β induced by ANIT in liver tissue (Zhao et al., 2017). Moreover, a study focusing on *Paeonia lactiflora* Pall., one of the sources of paeoniflorin, was in accordance with the previous result showing the suppression of the inflammatory response (Ma et al., 2018). Another study further revealed that paeoniflorin could mainly regulate primary bile acid biosynthesis by serum metabolomic profiling analysis (Chen et al., 2016)]. Therefore, transporters might be the central regulatory process. In 2017, Zhao reported that ANIT-induced dysregulated hepatocyte transporters, such as Na^+^/taurocholate-cotransporting polypeptide (NTCP), bile salt export pump (BSEP), and multidrug resistance-associated protein 2 (MRP2), were restored by paeoniflorin treatment (Zhao et al., 2017).

## Liver Fibrosis Attenuation

Liver fibrosis is the process of chronic liver injury caused by hepatitis B and C, alcohol consumption, fatty liver disease, cholestasis, and autoimmune hepatitis (Seki and Brenner, 2015). Hepatic stellate cell (HSC) activation plays a key role in myofibroblasts that produce extracellular matrix (ECM) in the liver (Tsuchida and Friedman, 2017). Currently, a variety of inflammatory and fibrogenic pathways are thought to participate in liver fibrosis (Seki and Schwabe, 2015; Higashi et al., 2017; Wree et al., 2018).

In the CCl_4_-induced liver fibrosis model, paeoniflorin was proven to effectively attenuate serum ALT, AST, HA, IV-C, and liver tissue Hyp at the doses of 20, 40, 80, and 200 mg/kg (Min et al., 2011). This result indicated that paeoniflorin could significantly decrease liver fibrosis development. Moreover, the inhibition of HIF-1α expression partly through the mTOR pathway might be the crucial mechanism (Zhao et al., 2014). This pharmacologic effect was also confirmed in two other liver fibrosis models. Hu reported that paeoniflorin treatment from 20 to 80 mg/kg for 26 consecutive weeks was able to inhibit radiation-induced hepatic fibrosis. The expression levels of TGF-β1, Smad3/4, and Smad7 were significantly lower in the paeoniflorin-treated groups than in the model group. This result indicated that paeoniflorin alleviated fibrosis *via* the TGF-β1/Smad signaling pathway (Hu et al., 2018). In addition to the radiation model, dimethylnitrosamine (DMN) was also used to induce liver fibrosis. Paeoniflorin treatment demonstrated an antifibrosis effect in rats with less collagen fiber deposition and gentle centrilobular necrosis observed in paeoniflorin-treated rats compared with DMN-induced model rats. These results were at least in part due to restored macrophage disruption and reduced inflammatory cytokines (Chen et al., 2012).

In addition, schistosomiasis is a kind of special chronic disease leading to liver fibrosis. A recent study demonstrated that paeoniflorin at 50 mg/kg/d improved parasitological parameters, such as decreased worm burden, immature eggs, and mature eggs, in a schistosomiasis *mansoni*-induced hepatic fibrosis model. Meanwhile, paeoniflorin treatment also significantly decreased the hepatic mean granuloma diameter and fibrosis area. The mechanism was partially recognized as targeting the apoptosis pathway by regulating caspase-3 and P53 expression (Abd El-Aal et al., 2017). Moreover, the key role of IL-13 was also explored in this model. Three other studies confirmed that paeoniflorin had a significant suppressive effect on the establishment of the ECM. Paeoniflorin could not only directly inhibit the alternative activation of macrophages by inhibiting the JAK2/STAT6 signaling pathway but also indirectly suppressed macrophages by decreasing IL-13 secretion (Li et al., 2009; Li et al., 2010; Chu et al., 2011).

## Nonalcoholic Fatty Liver Disease Prevention

Nonalcoholic fatty liver disease (NAFLD) has been the most common chronic liver disease worldwide in recent years. More than 40% of the population is affected in some countries. NAFLD has attracted concern worldwide since becoming a public health burden (Neuschwander-Tetri et al., 2010; Estes et al., 2018). NAFLD includes a wide range of liver disorders extending from nonalcoholic fatty liver (NAFL) to nonalcoholic steatohepatitis (NASH). Fibrosis, cirrhosis, and hepatocellular carcinoma will ultimately occur without treatment (Lebeaupin et al., 2018).

Paeoniflorin is a potential NAFLD prevention compound according to many studies. Zhang revealed that paeoniflorin attenuated NAFLD by restoring serum ALT, AST, TC, TG, HDL, and LDL. At the same time, paeoniflorin alleviated high-fat diet-induced hepatic adipose infiltration by decreasing steatosis, inflammation, ballooning degeneration, and necrosis. The potential mechanism might be cardiovascular protection by decreasing body weight and hyperlipidemia, blocking inflammation, and inhibiting lipid deposition (Zhang et al., 2015). Further research indicated that paeoniflorin ameliorated hepatic steatosis and inhibited CD68 and TGF-β1 expression. Downregulation of the ROCK/NF-κB signaling pathway might be relevant to the effect of paeoniflorin on NAFLD (Ma et al., 2016). Ma's investigation indicated that 20 mg/kg paeoniflorin remarkably inhibited lipid ectopic deposition *via* the lipid metabolism pathway. On the other hand, paeoniflorin treatment also exerted insulin sensitizing effects *via* IRS/Akt/GSK3β and antioxidation (Ma et al., 2017). In addition, a recent study also confirmed that paeoniflorin significantly reduced serum insulin and glucagon levels, enhanced insulin sensitivity, restored serum lipid profiles, and attenuated hepatic steatosis. All these effects should be relevant to the activation of the LKB1/AMPK and Akt signaling pathways in NAFLD (Li et al., 2018).

## Hepatocellular Carcinoma Inhibition

Hepatocellular carcinoma (HCC) is believed to be the most common and malignant type of tumor. HCC is the third most common cancer-related cause of death due to poor prognosis. Over 700,000 HCC cases are diagnosed every year (Robinson et al., 2019). The situation is particularly concerning in China. China accounts for 55% of HCC cases worldwide (Su et al., 2016). HCC is recognized to involve multiple signaling cascades in cell adhesion, cell migration, and extracellular matrix proteolysis (Brown and Murray, 2015). Therefore, potential agents for HCC treatment should efficiently address multiple aspects of this process.

In an *in vitro* study, paeoniflorin at the doses of 6.25–200 μM was found to significantly inhibit the growth of HepG2 and Bel-7402 cell lines. Proteolysis could reduce the invasion, metastasis, and adhesion of HCC cell lines. In addition, paeoniflorin was able to decrease MMP-9 and ERK levels and increase E-cad expression in HepG2 and Bel-7402 cells (Lu et al., 2014). Moreover, another study also indicated paeoniflorin as a promising agent in the treatment of liver cancer. Its mechanism might be partially related to apoptosis induction in hepatocellular carcinoma cells by downregulating prostaglandin E receptor EP_2_ levels, increasing the Bax/Bcl-2 ratio and thus upregulating the activation of caspase-3 (Hu et al., 2013).

## Outlook and Conclusion

**Figure 1:**
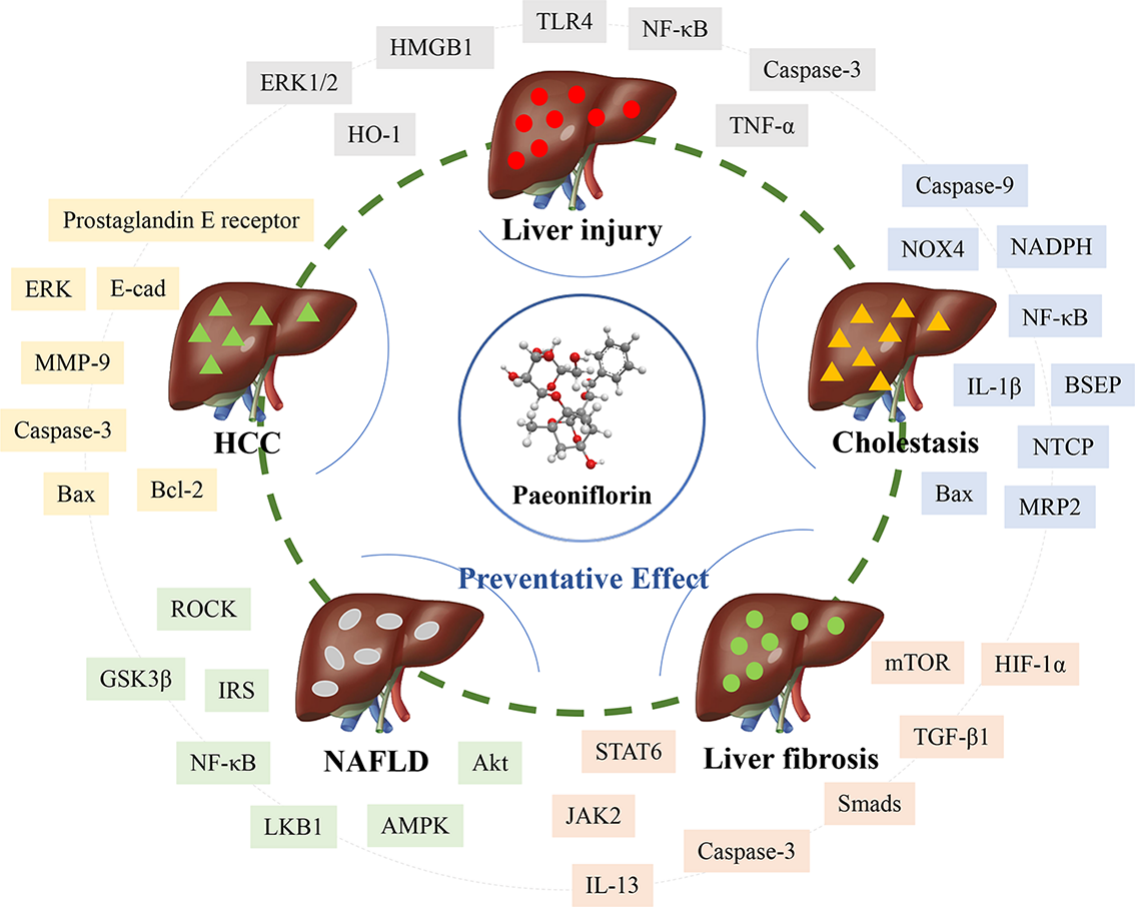
The pharmacological effect of paeoniflorin on liver diseases through multiple targets.

As evidenced by the numerous studies that have focused on the mechanism in-depth, many attempts have been made to investigate the efficacy of natural compounds such as paeoniflorin in liver disease treatment. This mini-review summarizes the pharmacologic activities and liver protection provided by paeoniflorin and demonstrates that paeoniflorin from the dosage of 5–200 mg/kg *in vivo* is an important compound for hepatic protection, cholestasis alleviation, liver fibrosis attenuation, NAFLD prevention, and HCC inhibition (**Table 1**). It is also crucial to reveal the mechanism to determine how paeoniflorin exerts its pharmacological effect. Paeoniflorin displays remarkable anti-inflammation effects *via* the TLR4-NF‐κB and ROCK/NF-κB signaling pathways during liver injury and NAFLD. Antioxidation signals such as HO-1, mitochondria-dependent pathways, and immune regulation containing HMGB1‐TLR4 are closely correlated. Moreover, paeoniflorin also alleviates cholestasis through an antioxidative mechanism by downregulating ROS and NOX4 and upregulating the PI3K/Akt/Nrf2 pathway. The anti-inflammatory effects of NF‐κB and IL-1β and the regulation of NTCP, BSEP, and MRP2 are mainly relevant to the anti-cholestatic effects of paeoniflorin. Several important signaling pathways, such as mTOR/HIF-1α, TGF-β1/Smads, and JAK2/STAT6, are involved in the effect of paeoniflorin on activated HSC and ECM inhibition during liver fibrosis. Macrophage regulation is also considered the crucial mechanism for the antifibrotic effect. The reduction in invasion, metastasis, and adhesion and the induction of apoptosis signals, including Bax, Bcl-2, and caspase-3, are related to the effect on hepatocellular carcinoma (**Figure 1**).

**Table 1:** The pharmacological activities of paeoniflorin in liver diseases.

Disease Treatment	Experimental model	Doses (Route)	Targets/Pathways	Reference
Liver injury	Hepatic I/R-induced injury	100 mg/kg (i.g.)	HMGB1-TLR4 pathway	Xie et al., 2018
	Hepatic I/R-induced injury	5–20 mg/kg (i.v.)	NF-κB signaling pathway and caspase-3	Tao et al., 2016
	CCl_4_-induced liver injury	10–100 mg/kg (i.g.)	HO-1, TNF-α, IL-6, and caspase-3	Guo et al., 2018
	Con A-induced liver injury	50 mg/kg (i.v.)	TLR4-NF-κB pathway	Chen M. et al., 2015
	Con A-treated HHSECs	50–800 μM (*in vitro*)	ERK1/2 and Akt phosphorylation	Gong et al., 2015
	GalN/TNF-α-treated L02	1–100 μM (*in vitro*)	ER stress and mitochondria-dependent pathway	Jiang et al., 2014
	BCG/LPS-induced immunological liver injury	25–100 mg/kg (i.g.)	TNF-α, IL-6, and LPS receptor	Liu et al., 2006
Cholestasis	ANIT-induced cholestasis	100–200 mg/kg (i.g.)	ROS-related NADPH and NOX4	Zhao et al., 2013
	ANIT-induced cholestasis	50–200 mg/kg (i.g.)	Apoptosis-related Bax, Caspase-9, and caspase-3	Zhou et al., 2017
	ANIT-induced cholestasis	50–200 mg/kg (i.g.)	PI3K/Akt/Nrf2 pathway	Chen Z. et al., 2015
	ANIT-induced cholestasis	50–200 mg/kg (i.g.)	NF-κB, IL-1β and the hepatic transporters NTCP, BSEP, and MRP2	Zhao et al., 2017
	ANIT-induced cholestasis	50–200 mg/kg (i.g.)	Primary bile acid biosynthesis	Chen et al., 2016
Liver fibrosis	CCl_4_-induced liver fibrosis	20–80 mg/kg (i.g.)	IV-C, LN, and Hyp reduction	Min et al., 2011
	CCl_4_-induced liver fibrosis	80–200 mg/kg (i.g.)	mTOR/HIF-1α signaling pathway	Zhao et al., 2014
	Radiation-induced liver fibrosis	20–80 mg/kg (i.g.)	TGF-β1/Smads signaling pathway	Hu et al., 2018
	DMN-induced liver fibrosis	20 mg/kg (i.g.)	Macrophage disruption	Chen et al., 2012
	Schistosomiasis *mansoni*-induced liver fibrosis	50 mg/kg (i.g.)	Apoptosis pathway related to caspase-3 and P53	Abd El-Aal et al., 2017
	Schistosomiasis *japonica*-induced liver fibrosis	60 mg/kg (i.g.)	JAK2/STAT6 signaling pathway and IL-13	Chu et al., 2011
	Schistosomiasis *japonica*-induced liver fibrosis/Hepatic stellate cells	30 mg/kg (i.g.) /30–120 mg/L (*in vitro*)	SOCS-1, STAT6, and IL-13	Li et al., 2010
	Schistosomiasis *japonica*-induced liver fibrosis	30 mg/kg (i.g.)	IL-13 and IL-13Ra2	Li et al., 2009
NAFLD	AIN76A diet-induced NAFLD	0.05% (in diet)	Lipid synthesis, inflammation, and hyperglycemia pathway	Zhang et al., 2015
	HCF diet-induced NAFLD	20–100 mg/kg (i.g.)	ROCK/NF-κB signaling pathway	Ma et al., 2016
	2% cholesterol and 15% lard diet-induced NAFLD	20 mg/kg (i.g.)	IRS/Akt/GSK3β, antioxidation, and insulin sensitizing	Ma et al., 2017
	Fructose-induced insulin resistance and hepatic steatosis	10–40 mg/kg (i.g.)	LKB1/AMPK and Akt signaling pathway	Li et al., 2018
HCC	Human HCC Bel-7402 and HepG2 cell lines	6.25–200 μM (*in vitro*)	MMP-9, ERK, and E-cad	Lu et al., 2014
	Human HCC HepG2 and SMMC-7721 cell lines	10^−8^–10^−5^ mol/L (*in vitro*)	Prostaglandin E receptor EP2, Bax, Bcl-2, and caspase-3	Hu et al., 2013

ANIT, alpha-naphthylisothiocyanate; BCG, bacillus Calmette-Guérin; CCl_4_, carbon tetrachloride; Con-A, concanavalin A; DMN, dimethylnitrosamine; HCC, hepatocellular carcinoma; HCF, High-fat; HHSECs, human hepatic sinusoidal endothelial cells; i.g., intragastric; I/R, ischemia/reperfusion; i.v., intravenous injection; LPS, lipopolysaccharide; NAFLD, Nonalcoholic fatty liver disease.

The common mechanisms could be summarized from the current literature. The anti-inflammation, anti-oxidative and anti-apoptosis in hepatocytes are the core functions for its effect on liver diseases. Moreover, the immune and macrophage regulation are also important for its special effect on liver damage and liver fibrosis. The effect of paeoniflorin on liver diseases has been vastly developed, accompanied by deep insight into mechanistic investigation. Even so, two essential aspects ought to be noted based on this mini-review. First, most of the signals mentioned above are the downstream targets in various liver diseases. The upstream of signals which paeoniflorin targets directly will be drawn with special attention. Second, particular focus should also be paid to the ‘from bench to bedside' concept for further clinical discovery. The clinical conversion with rigorous randomized controlled trial is the golden index to check the efficacy and medicinal value of paeoniflorin. Therefore, the deeper mechanistic investigation and the further clinical confirmation seem as the two key processes in the future development.

In summary, paeoniflorin demonstrates multiple effects on liver diseases correlating with complex and complicated signaling pathways. Therefore, paeoniflorin might be a potential agent to treat liver disease and alleviate liver damage.

## Author Contributions

XM, WZ, and YJ prepared the manuscript. JW and SW reviewed the drafts and provided important information for the completion. YZ conceived the idea and provided important information for the completion.

## Funding

This work was supported by National Natural Science Foundation of China (81874365), Sichuan Science and Technology Program (2019YJ0492), China Postdoctoral Science Found Grant (2017M622987), and Chengdu University of TCM Found Grant (QNXZ2018025).

## Conflict of Interest

The authors declare that the research was conducted in the absence of any commercial or financial relationships that could be construed as a potential conflict of interest.

## Abbreviations

ALP, Alkaline phosphatase. ANIT, Alpha-naphthylisothiocyanate. BCG, Bacillus Calmette-Guérin. BSEP, Bile salt export pump. CCl_4_, Carbon tetrachloride. Con-A, Concanavalin A. DMN, Dimethylnitrosamine. ECM, Extracellular matrix. GalN, D-galactosamine. LPS, lipopolysaccharide. GSH, Glutathione. GCLc, Glutamate-cysteine ligase catalytic subunit. GCLm, Glutamate-cysteine ligase modifier subunit. HCC, Hepatocellular carcinoma. HHSECs, Primary human hepatic sinusoidal endothelial cells. HSCs, Hepatic stellate cells. I/R, Ischemia/reperfusion. MRP2, Multidrug resistance-associated protein 2. NAFL, Nonalcoholic fatty liver. NAFLD, Nonalcoholic fatty liver disease. NASH, Nonalcoholic steatohepatitis. NTCP, Na^+^/taurocholate-cotransporting polypeptide. TBA, Total bile acid. γ-GT, γ-Glutamyltranspeptidase. i.g., Intragastric. i.v., Intravenous injection.
